# Diagnostic Accuracy of Transvaginal Ultrasonography for Endometriosis According to the International Deep Endometriosis Analysis Consensus

**DOI:** 10.1097/og9.0000000000000061

**Published:** 2025-02-20

**Authors:** Ido Mick, Shay M. Freger, Melissa Marien, Mahsa Gholiof, Mathew Leonardi

**Affiliations:** Department of Obstetrics & Gynecology, McMaster University, Hamilton, Ontario, Canada; and Robinson Research Institute, University of Adelaide, Adelaide, Australia.

## Abstract

**OBJECTIVE:**

To evaluate the diagnostic accuracy of transvaginal ultrasonography (TVUS) in detecting deep endometriosis, ovarian endometriosis, and superficial endometriosis using the IDEA (International Deep Endometriosis Analysis) consensus and novel International Terminology definitions for endometriosis phenotypes.

**METHODS:**

This prospective diagnostic accuracy study was conducted at McMaster University from November 2021 to January 2023. Participants included 125 individuals aged 18–50 years with suspected endometriosis who were undergoing TVUS followed by laparoscopic surgery. The index test, TVUS, was performed in accordance with the IDEA consensus, with results compared with laparoscopy and histopathologic findings, which served as the reference standard. *Deep endometriosis* was defined as endometriosis with any depth of infiltration, and *superficial endometriosis* was defined as endometriosis without any infiltration beyond the peritoneal surface. Diagnostic accuracy metrics, including sensitivity, specificity, positive predictive value (PPV), and negative predictive value were calculated.

**RESULTS:**

The study demonstrated high diagnostic accuracy for endometriomas and deep endometriosis with TVUS, achieving a sensitivity of 95.0% for left ovarian endometrioma, 94.9% for right ovarian endometrioma, and sensitivity ranging from 84.3% to 100% for various deep endometriosis sites. Transvaginal ultrasonography showed high specificity for endometriomas (98.9%) and deep endometriosis (range 97.0–100%). However, TVUS showed lower sensitivity for superficial endometriosis (range 4.0–43.5%) but high specificity (99.0–100%) and PPV (90.0–100%). The accuracy of detecting superficial endometriosis improved when cases with ovarian endometriosis, deep endometriosis, and pouch of Douglas obliteration were excluded.

**CONCLUSION:**

Transvaginal ultrasonography demonstrates robust diagnostic accuracy for deep endometriosis and ovarian endometriosis, reaffirming its utility as a first-line diagnostic tool in endometriosis management despite changes in definitions of deep endometriosis and superficial endometriosis. However, its sensitivity for superficial endometriosis remains limited, suggesting the need for additional diagnostic techniques or refined ultrasound approaches for accurate detection of superficial endometriosis. The findings support the integration of TVUS in clinical practice while highlighting the need for further advancements in diagnosing superficial endometriosis to mitigate diagnostic delays.

## Abstract 2

Transvaginal ultrasonography demonstrates high diagnostic accuracy for deep and ovarian endometriosis using updated terminology, but sensitivity for superficial endometriosis remains limited.

Endometriosis affects 10% of all people assigned female at birth^1^ and is characterized by the presence of endometrial-like tissue outside the uterus, resulting in chronic inflammation, pelvic pain, and infertility.^2^ Despite the numerous challenges in endometriosis research and clinical care, one of the most significant issues is the considerable diagnostic delay associated with the disease. This delay leads to substantial negative effects on quality of life and increased health care resource utilization and costs.^3,4^ The diagnostic delay associated with endometriosis is likely multifactorial, including limited awareness of endometriosis, normalization of symptoms, and the underutilization of presumptive diagnoses in the absence of clear imaging.^3–6^ The difficulty in detecting superficial endometriosis without accompanying deep endometriosis or endometriomas further complicates timely diagnosis.^5,6^

Historically, surgical visualization through laparoscopy followed by histologic confirmation was the most widely adopted method for diagnosing endometriosis.^7^ However, with recent advancements, including the recent Society of Radiologists in Ultrasound consensus^8,9^ and European Society of Human Reproduction and Embryology guidelines (and several others), transvaginal ultrasonography (TVUS) is fully recognized as the first-line diagnostic for endometriosis.^10,11^ Recent studies have illuminated the high diagnostic accuracy of TVUS, particularly for endometriomas, deep endometriosis, and adhesion states such as pouch of Douglas (also known as rectouterine pouch or cul-de-sac) obliteration.^12,13^ However, superficial endometriosis, the most common phenotype of endometriosis, present in 80% of all patients diagnosed, remains an elusive target when using noninvasive modalities,^5,14^ though ultrasonographic features have been described previously.^5,14^

In 2016, the IDEA (International Deep Endometriosis Analysis) group published a pioneering consensus to standardize ultrasound evaluation methods and nomenclature for diagnosing endometriosis.^15^ Later, in 2021, a collaborative “International Terminology” consensus aimed to standardize nomenclature for endometriosis phenotyping,^16^ modifying the definition of superficial endometriosis from “endometriosis <5 mm of infiltration beneath the peritoneal surface” to “endometriosis without any infiltration beyond the surface.” Notably, this also changed deep endometriosis to mean endometriosis with any degree of infiltration. Although it is positive that we have seen prospective studies of the IDEA approach,^6,17,18^ these unfortunately occurred before the International Terminology consensus or did not incorporate the new definitions, calling into question the modern relevance of their interpretation. Because deep endometriosis may be physically smaller (or at least less deep in infiltration) now with the new definition, previous results may overestimate the higher diagnostic performance of TVUS (and other imaging tests) for deep endometriosis.

This study aims to estimate and compare the diagnostic accuracy of TVUS reported in accordance with the IDEA consensus and novel International Terminology definitions for all phenotype presentation of endometriosis, including deep endometriosis, ovarian endometriosis, and superficial endometriosis. Our hypothesis posited that TVUS would have a reduced diagnostic performance for deep endometriosis using the novel classification guidelines, when compared against published literature, with the inclusion of smaller nodules (less than 5 mm) that would have historically been considered superficial endometriosis.

## METHODS

This study is performed and reported according to STARD (Standards for Reporting Diagnostic Accuracy) guidelines^19^ to assist in standardization and transparency of reporting diagnostic accuracy. The Quality Assessment of the Diagnostic Accuracy of Studies checklist was adopted to reduce risk of bias throughout study methodology, including patient selection, index and reference tests, and flow and timing.^20^

This is a diagnostic test accuracy study completed at McMaster University Medical Center, Hamilton Health Sciences, Canada. Participant recruitment spanned from November 2021 to January 2023. This study was approved by the Hamilton Integrative Research Ethics Board (HiREB: 12617).

Consecutive participants were prospectively recruited and evaluated if they met the inclusion criteria. Inclusion criteria included age between 18 and 50 years, assigned female sex at birth, premenopausal and postmenarchal, history of chronic pelvic pain or endometriosis, able to undergo TVUS, and consented to laparoscopic surgery for endometriosis. Exclusion criteria included suspected or confirmed pelvic malignancy (based on medical history and prior imaging reports), premenarchal, menopausal, suspected or confirmed pregnancy, and surgery performed more than 1 year after an ultrasound scan. Participant variables, which included age, gravidity, parity, body mass index (BMI, calculated as weight in kilograms divided by height in meters squared), symptoms associated with endometriosis, and previous surgery for endometriosis, were documented.

The index test, TVUS, was performed and reported in accordance with the IDEA consensus with the additional posterior approach technique discussed by Leonardi et al^21^ for the evaluation of deep endometriosis of the uterosacral ligaments and torus uterinus. Transvaginal ultrasonography was conducted within 1 year of the scheduled surgical date, with a repeat scan performed on the day of surgery to ensure no changes in disease state, with index test findings updated accordingly before surgery. All TVUS scans were performed and reported by a single, highly experienced surgeon–sonologist (M.L.), based on the European Federation of Societies for Ultrasound in Medicine and Biology (level 3). The International Terminology definitions^16^ were used throughout this study. In the assessment of the ovaries, endometriomas was classified by cystic lesions with ground glass echogenicity. When suspected on TVUS, superficial endometriosis was classified in line with Leonardi et al^5^ previous work by hyperechoic foci, cystic, interrupted along the peritoneal surface, or velamentous (filmy) adhesions along the peritoneum. In the presence of physiologic fluid within the pouch of Douglas, superficial endometriosis was assessed by inducing mobility of the fluid using the “jiggle test,” which includes applying pressure of the probe or abdomen, to confirm its adherence to the peritoneal surface rather than debris (eg, blood) that might simply be settled (Video 1). Patients without pelvic peritoneal fluid could not be assessed for superficial endometriosis, as the absence of fluid limits the ability of TVUS to delineate peritoneal surfaces and identify superficial endometriosis. Moreover, in cases of severe deep endometriosis and the obliteration of the pouch of Douglas, assessment of posterior compartment superficial endometriosis was not possible and not done. The presence of pouch of Douglas obliteration was assessed using the “sliding sign” method^22^ to assess the mobility of the uterus. Mindray Zonare machines were used in 2021 with E9–4 (4–9 MHz) transducers, followed by a transition to a GE Healthcare Voluson E10 machines with either 5–9 MHz or 6–12 MHz for the remainder of this study.

The index test was compared with histology after laparoscopy. All surgeries were performed within 1 year from the index test. Direct visualization involved a systematic evaluation of the abdomen and pelvis of all surfaces. The surgeon (M.L.) was not blinded to the ultrasound findings, which reflects the real clinical experience of surgeons who use preoperative information to plan and perform surgery appropriately. Pathologists were blinded to the index test. Laparoscopic excision of endometriosis was complete, except in cases where the patient preferred conservative management of, specifically, bowel endometriosis.

The entire pelvic cavity was assessed, mirroring the systematic ultrasound evaluation approach. The following sites were assessed: bilateral ovaries, posterior and anterior aspects of the uterus, bladder, vesicouterine peritoneum, pelvic sidewalls uterosacral ligaments, torus uterinus, posterior vaginal fornix, and bowel. The presence of deep endometriosis was classified when irregular nodules of varying pigmentations with fibrotic (or hard) tactile feedback on palpation were noted, requiring dissection and excision of tissue beneath the surface of the peritoneum. An endometrioma was noted when an ovarian cyst containing dark brown material (“chocolate cyst”) was present. Superficial endometriosis was noted as a red, white, clear, or blue–black deposit along the peritoneal surface, dissectible via wide peritonectomy or ablation without the need to excise tissue below the peritoneal surface. Each anatomical site assessed intraoperatively was reported as either positive or negative for deep endometriosis, ovarian endometriosis, or superficial endometriosis. All tissue was excised and sent to pathology for blinded assessment by a single experienced pathologist, including the presence of endometrial glands or stroma in removed tissue. Obliteration of the pouch of Douglas was noted when the peritoneum between and inferior to the uterosacral ligaments was not visible or present due to the presence of adhesions. Partial obliteration of the pouch of Douglas was denoted by partial adhesions in this space, permitting some visualization of the peritoneum.

The sample size for the TVUS technique was determined based on Buderer's formula, a statistical method used to estimate the sample size required for diagnostic accuracy studies, accounting for disease prevalence, expected sensitivity and specificity, and the desired confidence level.^23^ Using our current prevalence of surgically confirmed superficial endometriosis within our clinical population of 70% and pooled expected sensitivity and specificity among all sites of superficial endometriosis of 35% and 95%, respectively (confidence level of 95%; 0.90 power), a total of 125 participants was required.

Data were collected in real-time using the REDCap electronic data-capture tool (Vanderbilt University, Nashville, Tennessee) and were imported into Microsoft Excel for Windows 10. Data were cleaned to ensure accuracy and consistency before analysis. This included reviewing and coding for missing values, correcting data-entry errors, standardizing variable formats, and verifying logical consistency across variables (eg, aligning surgical and ultrasound findings where applicable). Finalized data were transferred and analyzed using IBM SPSS statistics V29 software.

Descriptive statistics were used to summarize all variables. Continuous variables were described with means and respective standard deviations. Categorical variables were described as frequencies and percentages. Additional variables identified during TVUS or laparoscopy with histologic confirmation, including endometriosis phenotypes, were reported as frequencies and percentages relative to the total population.

Accuracy, sensitivity, specificity, negative predictive value (NPV), positive predictive value (PPV), negative likelihood ratio, and positive likelihood ratio with 95% CI were calculated for the index test relative to laparoscopy as the primary reference standard among all participants. All accuracy parameters were determined using the cross-tabulation function in SPSS.

## RESULTS

**Fig. 1.**
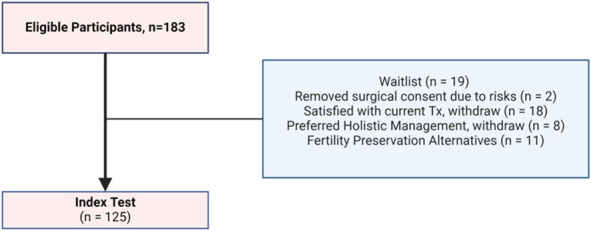
Study flow chart depicting participant inclusion and exclusion. Tx, treatment.

A total of 183 potential participants were screened for eligibility; 125 participants met the inclusion criteria and underwent the index test and surgery within the date range. The flow of participants, including inclusion and exclusion, is reported in Figure 1. Participant characteristics are reported in Table 1.


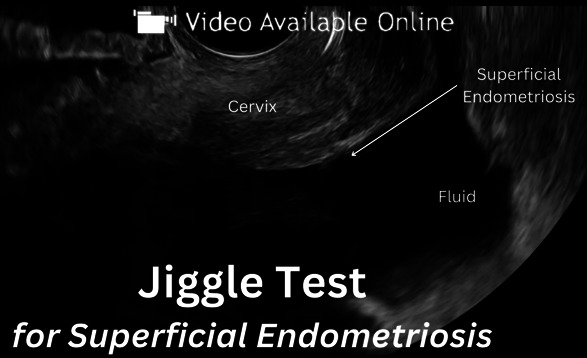
 **Video 1.** Demonstration of the jiggle test for detecting superficial endometriosis. This technique involves applying gentle pressure with the transvaginal ultrasonography probe to induce movement of pelvic fluid, allowing visualization of superficial endometriosis lesions along the peritoneal surface. Video created by Ido Mick, Shay M. Freger, Melissa Marien, Mahsa Gholiof, and Mathew Leonardi. Used with permission.

*Mick. Transvaginal Ultrasonography for Endometriosis. O&G Open 2025*.

**Table 1.** Characteristics of Included Participants (N=125)

Characteristic	Value
Demographics	
Age (y)	36.6±6.8
Gravidity	
0	56
1	23
2	18
3 or more	28
Parity	
0	77
1	18
2	20
3 or more	10
BMI (kg/m^2^)	27.0 (±6.1)
Previous surgery and management	
Previous endometriosis surgery	28.8 (36)
Previous cesarean delivery	14.4 (18)
Hormonal therapy	40.0 (50)
Symptoms	
Dysmenorrhea	85.6 (107)
Chronic pelvic pain	71.2 (89)
Dyspareunia	59.2 (74)
Dyschezia	53.6 (67)
AUB	44.0 (55)
Infertility	30.4 (38)
Dysuria	23.2 (29)
Hematochezia	8.8 (11)
Hematuria	0.8 (1)

BMI, body mass index; AUB, abnormal uterine bleeding.

Data are mean±SD, n, or % (n).

The most frequent locations for histologically confirmed deep endometriosis were found to be the uterosacral ligaments 85.6% (107/125), torus uterinus 23.2% (29/125), bowel 18.4% (23/125), and posterior vaginal fornix 12.8% (16/125). There was no deep endometriosis of the bladder in our study, both surgically and ultrasonographically. An endometrioma was seen in the left and right ovaries in 33.3% (40/120) and 31.4% (39/124) of patients, respectively. Among patients with assessable pouch of Douglas obliteration state, 33.9% (42/124 patients) had obliteration of the pouch of Douglas laparoscopically, with one patient not accessible due to a previous hysterectomy. Left endometriomas were not assessable in the five patients, and right endometriomas were not assessable in one patient. These patients had undergone previous oophorectomies. The torus uterinus and bilateral uterosacral ligaments were not assessable in two patients due to complex disease circumvention of ultrasonographic and laparoscopic visualization.

Among all participants, the left pelvic sidewall had the highest prevalence of laparoscopically visualized and histologically confirmed superficial endometriosis, with a prevalence of 40.0% (50/125). This was followed by pouch of Douglas 28.0% (35/125), posterior uterine or cervix serosa 19.2% (24/125), right uterosacral ligament 19.2% (24/125), left uterosacral ligament 18.4% (23/125) and right pelvic sidewall 18.4% (23/125).

The diagnostic performance of TVUS in diagnosing deep endometriosis and endometriomas is reported in Table 2. The diagnostic performance of TVUS in diagnosing endometriomas of the left and right ovaries was as follows: accuracy 98.3% (95% CI, 94.1–99.8%) for the left and 98.4% (95% CI, 94.3–99.8%) for the right, sensitivity 95.0% (95% CI, 83.1–99.4%) for the left and 94.9% (95% CI, 82.7–99.4%) for the right, specificity 100% (95% CI, 95.5–100%) for the left and 100% (95% CI, 95.8–100%) for the right, PPV 100% (95% CI, 90.7–100%) for the left and 100% (95% CI, 90.5–100%) for the right, and NPV 97.6% (95% CI, 91.2–99.4%) for the left and 97.7% (95% CI, 91.8–99.4%) for the right. In the assessment of deep endometriosis among all sites, the diagnostic performance ranges of TVUS were as follows: accuracy 93.5–99.2%, sensitivity 84.3–100%, specificity 97.0–100%, PPV 94.1–100%, and NPV 90.0–100%. In the assessment of pouch of Douglas obliteration, the performance was as follows: accuracy 97.6% (95% CI, 93.0–99.5%), sensitivity 97.6% (95% CI, 87.4–99.9%), specificity 97.5% (95% CI, 91.4–99.7%), PPV 95.4% (95% CI, 83.9–98.8%), and NPV 98.7% (95% CI, 91.8–99.8%).

**Table 2.** Diagnostic Accuracy Parameters of Transvaginal Ultrasonography Relative to Laparoscopy as the Reference Standard for Ovarian Endometriosis and Deep Endometriosis

	Left Ovary	Right Ovary	Left USL	Right USL	TU	Posterior Vaginal Fornix	Bowel	POD Obliteration
Accuracy	98.3 (94.1–99.8)	98.4 (94.3–99.8)	93.5 (87.6–97.1)	93.5 (87.6–97.1)	97.6 (93.0–99.5)	99.2 (95.6–100)	98.4 (94.3–99.8)	97.6 (93.0–99.5)
Sensitivity	95.0 (83.1–99.4)	94.9 (82.7–99.4)	89.3 (78.1–96.0)	84.3 (71.4–93.0)	93.1 (77.2–99.1)	100 (79.4–100)	95.6 (78.0–99.9)	97.6 (87.4–99.9)
Specificity	100 (95.5–100)	100 (95.8–100)	97.0 (89.6–99.6)	100 (95.1–100)	98.9 (94.2–100)	99.1 (95.0–100)	99.0 (94.6–100)	97.5 (91.4–99.7)
PPV	100 (90.7–100)	100 (90.5–100)	96.1 (86.4–98.8)	100 (91.6–100)	96.4 (79.3–99.5)	94.1 (69.5–99.1)	95.6 (75.7–99.4)	95.4 (83.9–98.8)
NPV	97.6 (91.2–99.4)	97.7 (91.8–99.4)	91.5 (83.5–95.8)	90.0 (82.6–94.4)	97.9 (92.4–99.4)	100 (96.6–100)	99.0 (93.6–99.8)	98.7 (91.9–99.8)
LR+	—	—	29.9 (7.6–117.5)	—	87.5 (12.4–616.4)	109.0 (15.5–766.9)	96.6 (13.7–680.5)	39.1 (9.9–153.6)
LR−	0.05 (0.01–0.2)	0.05 (0.01–0.2)	0.1 (0.05–0.2)	0.1 (0.08–0.3)	0.07 (0.02–0.3)	—	0.04 (0.01–0.3)	0.02 (0–0.2)

USL, uterosacral ligament; TU, torus uterinus; POD, pouch of Douglas; LR, likelihood ratio; PPV, positive predictive value; NPV, negative predictive value.

Data are % (95% CI).

In the assessment of superficial endometriosis among all patients and all sites (Table 3), the diagnostic performance ranges were as follows: accuracy 61.6–88.8%, sensitivity 4.0–43.5%, specificity 99.0–100%, PPV 90.0–100%, and NPV 61.0–88.7%. After excluding patients with ovarian endometriosis, deep endometriosis, and pouch of Douglas obliteration (Table 4), the performance ranges were as follows: accuracy 74.4–88.4%, sensitivity 15.4–50.0%, specificity 100%, PPV 100%, and NPV 72.7–84.6%. Overall, the right uterosacral ligament had the highest overall accuracy (88.4%, 95% CI, 74.9–96.1%), followed by the right pelvic sidewall (88.4%, 95% CI, 74.9–96.1%), the left uterosacral ligament (81.4%, 95% CI, 66.6–91.6%), the pouch of Douglas (79.1%, 95% CI, 64.0–90.0%), and the left pelvic sidewall (74.4%, 95% CI, 58.8–86.5). There were no patients with detectable superficial endometriosis of the vesicouterine and both aspects of the uterine serosa after excluding those with pouch of Douglas obliteration, deep endometriosis, and ovarian endometriosis. Counts for all phenotypes analysis are reported in Appendices 1–3, available at http://links.lww.com/AOG/D998.

**Table 3.** Diagnostic Accuracy Parameters of Transvaginal Ultrasonography Relative to Laparoscopy as the Reference Standard for Superficial Endometriosis in the Whole Population

	Vesicouterine	Uterus (Anterior)	Uterus (Posterior)	POD	Left PSW	Right PSW	Left USL	Right USL
Accuracy	84.0 (75.5–89.3)	88.8 (81.9–93.7)	82.4 (74.6–88.6)	81.6 (73.7–88.0)	61.6 (52.5–70.2)	83.1 (75.3–89.2)	88.8 (81.9–93.7)	87.2 (80.0–92.5)
Sensitivity	4.8 (0.1–23.8)	6.7 (0.2–31.9)	8.3 (1.0–27.0)	37.1 (21.5–55.1)	4.0 (0.5–13.7)	8.7 (1.1–28.0)	43.5 (23.2–65.5)	37.5 (18.8–59.4)
Specificity	100 (96.5–100)	100 (96.7–100)	100 (96.4–100)	99.0 (94.0–100)	100 (95.2–100)	100 (96.4–100)	99.0 (94.7–100)	99.0 (94.6–100)
PPV	100 (2.5–100)	100 (2.5–100)	100 (15.8–100)	92.9 (63.9–99.0)	100 (15.8–100)	100 (15.8–100)	90.9 (57.4–98.7)	90.0 (54.5–98.5)
NPV	83.1 (83.9–84.3)	88.7 (87.3–90.0)	82.1 (80.3–83.8)	80.2 (75.8–83.9)	61.0 (59.6–62.3)	82.8 (80.9–84.5)	88.6 (84.4–91.8)	87.0 (83.0–90.1)
LR+	—	—	—	33.4 (4.5–246.1)	—	—	44.35 (6.0–329.4)	37.9 (5.0–284.8)
LR−	0.9 (0.9–1.1)	0.9 (0.8–1.1)	0.9 (0.8–1.0)	0.6 (0.5–0.8)	1.0 (0.9–1.0)	0.9 (0.8–1.0)	0.6 (0.4–0.8)	0.6 (0.5–0.9)

POD, pouch of Douglas; PSW, pelvic sidewall; USL, uterosacral ligament; PPV, positive predictive value; NPV, negative predictive value; LR, likelihood ratio.

Data are % (95% CI).

**Table 4.** Diagnostic Accuracy Parameters of Transvaginal Ultrasonography Relative to Laparoscopy as the Reference Standard for Superficial Endometriosis, Excluding Patients With Pouch of Douglas Obliteration, Deep Endometriosis, and Ovarian Endometriosis

	POD	Left PSW	Right PSW	Left USL	Right USL
Accuracy	79.1 (64.0–90.0)	74.4 (58.8–86.5)	88.4 (74.9–96.1)	81.4 (66.6–91.6)	88.4 (74.9–96.1)
Sensitivity	35.7 (12.8–64.9)	15.4 (1.9–45.5)	16.7 (0.4–64.1)	33.3 (9.9–65.1)	50.0 (18.7–81.3)
Specificity	100 (88.1–100)	100 (88.4–100)	100 (90.5–100)	100 (88.8–100)	100 (89.4–100)
PPV	100 (47.8–100)	100 (15.8–100)	100 (2.5–100)	100 (39.8–100)	100 (47.8–100)
NPV	76.3 (68.6–82.6)	72.7 (68.4–77.5)	84.4 (83.8–91.4)	79.5 (72.2–85.2)	84.6 (78.0–92.5)
LR+	—	—	—	—	—
LR−	0.6 (0.4–1.0)	0.8 (0.7–1.1)	0.8 (0.6–1.2)	0.7 (0.4–1.0)	0.5 (0.3–0.9)

POD, pouch of Douglas; PSW, pelvic sidewall; USL, uterosacral ligament; PPV, positive predictive value; NPV, negative predictive value; LR, likelihood ratio.

Data are % (95% CI).

## DISCUSSION

In this diagnostic accuracy study, we aimed to evaluate the diagnostic performance of a standard TVUS in identifying deep endometriosis, ovarian endometriosis, and superficial endometriosis, reported per the IDEA consensus and novel International Terminology definitions for deep endometriosis and superficial endometriosis. Considering the inclusion of former superficial endometriosis (smaller-in-size nodules) in the phenotype category of deep endometriosis based on the new definitions, we hypothesized a reduced diagnostic performance of TVUS in diagnosing deep endometriosis, relative to previous literature.

Instead, our findings indicate an enhanced diagnostic accuracy in detecting endometriomas and deep endometriosis, compared with prior studies that similarly used the IDEA consensus, rejecting our initial hypothesis. The results highlight a high diagnostic specificity, affirming the utility of TVUS in accurately diagnosing all phenotypes of endometriosis, especially for definitively confirming the presence of all endometriosis phenotypes. Regarding the visualization of superficial endometriosis, efforts are needed to enhance the sensitivity of the test to adequately rule out disease.

Recent advancements in the field have led to novel guidelines recommending implementing TVUS as a first-line diagnostic tool for endometriomas and deep endometriosis,^24^ including the first North American organizations supporting ultrasonography for endometriosis.^8,9^ Although this is positive, most guidelines were developed using the historical classifications of deep endometriosis and superficial endometriosis. Considering the novel International Terminology definition of deep endometriosis incorporating nodules of less than 5 mm in depth, we hypothesized that the diagnostic accuracy may be reduced, which may urge more caution in the reliance on TVUS. Reassuringly, our findings suggest a maintained and even improved accuracy among all anatomical sites with the novel deep endometriosis definition. This may be partly secondary to the academic and clinical evolution to understand and assess all forms and locations of endometriosis, including superficial endometriosis, ultrasonographically.^5,6^

Indeed, alongside the diagnostic performance of endometriomas and deep endometriosis, this study demonstrates the potential of TVUS in diagnosing superficial endometriosis when present. Although our findings suggest an overall low sensitivity, TVUS showed a high specificity. After excluding patients with ovarian endometriosis, deep endometriosis, and pouch of Douglas obliteration, our results suggest an improvement in accuracy, with a specificity and PPV of 100% among all remaining sites, demonstrating the reliability of TVUS to rule in superficial endometriosis when visualized. This improvement is likely attributed to the reduced diagnostic complexity when ovarian endometriosis, deep endometriosis, and pouch of Douglas obliteration are excluded, which often lead to significant anatomical distortion and obscure visualization of subtle superficial endometriosis lesions. Our study corroborates the findings of a recently published study by Bailey et al,^6^ which reports TVUS correctly diagnosing superficial endometriosis with a sensitivity of 51.5% (33.5–69.2%) and specificity of 94.0% (85.4–98.4%). Importantly, the study by Bailey et al relied on the older definitions of superficial endometriosis and deep endometriosis, meaning that many lesions characterized as superficial endometriosis were instead technically deep endometriosis. A novel approach proposed by Leonardi et al^14^ using sonoPODography, whereby exogenous fluid was introduced into the pouch of Douglas to improve visualization of superficial endometriosis in accordance with the novel guidelines, suggested a sensitivity of 64.9% and specificity of 100%. After excluding patients with deep endometriosis, ovarian endometriosis, and pouch of Douglas obliteration, there was an improvement in parameters of sensitivity 77.7% and specificity 100%.

It is important to highlight that this study, and both previous studies, revealed the limitations of TVUS in effectively ruling out superficial endometriosis ultrasonographically. Furthermore, the importance of physiologic or exogenous fluid within the pelvis in aiding the diagnosis of superficial endometriosis has been appreciated,^14^ with novel techniques suggesting improved accuracy with the introduction fluid within the pouch. Indeed, the presence or absence of physiologic fluid in this current study supported or hindered our ability to visualize and characterize superficial endometriosis.

This study reinforces the significant role of TVUS as an effective diagnostic tool for deep endometriosis and ovarian endometriosis, offering clinicians a reliable early detection and management method. However, the limited sensitivity for superficial endometriosis highlights a potential shortfall in current clinical practice, where cases of superficial endometriosis might be underdiagnosed or missed entirely. This limitation may be attributed to the subtle and diffuse nature of superficial endometriosis lesions, which are challenging to detect with conventional TVUS techniques. The hypothesized mechanism underlying the reduced sensitivity for superficial endometriosis could involve the variability in lesion presentation, including differences in lesion size, infiltration depth, location within the pelvic cavity, or the presence or absence of physiologic fluid. These findings suggest that, although TVUS remains a valuable diagnostic tool, clinicians should exercise caution when ruling out superficial endometriosis and remain vigilant for superficial endometriosis in patients with negative TVUS results but persistent symptoms suggestive of endometriosis.

This study was performed in accordance with the IDEA consensus statement, with modern International Terminology definitions of deep endometriosis and superficial endometriosis used. Moreover, every effort was dedicated to fortifying the integrity of this study, meticulously adhering to the STARD guidelines^19^ and QUADAS-2 to reduce biases.^20^ Adopting a prospective design, we employed standardized reporting forms to ensure a seamless and consistent approach across consecutive patients.

Although this study upholds methodologic consistency and strengths, it is imperative to acknowledge that it is not without its limitations. The study was conducted by a highly trained surgeon-sonologist, which may influence the diagnostic accuracy and generalizability of the results. Additionally, all resected samples suspected of disease underwent pathologic assessment for endometriosis with a blinded pathologist against the index test TVUS; however, healthy tissue is seldom resected, limiting our ability to rule-out disease definitively using histology in tissue that is not resected. Our study employed a pragmatic approach where the surgeon's intraoperative classification of “normal” was accepted as true (ie, disease was absent). This decision aligns with past literature^7^ indicating no significant statistical differences when comparing laparoscopic and histologic modalities. However, it is crucial to clarify that the surgeon's assessment was not merely for validating endometriosis presence but was based on stringent surgical criteria that met established standards for endometriosis identification and excision. Additionally, the improved performance of TVUS observed in this study may also be attributable to the increased experience and expertise of the observer. External validation and larger-scale multicenter diagnostic accuracy studies are needed to fortify and expedite advancements in endometriosis diagnosis. Lastly, the study population, preselected based on clinical suspicion of endometriosis, reflects the diagnostic practices of a tertiary care endometriosis center. Although this enhances the relevance to specialized settings, it may limit generalizability to broader gynecologic populations.

Endometriosis continues to pose significant negative effects, primarily attributed to the diagnostic delay. With recent updates to deep endometriosis and superficial endometriosis definitions and growing guidelines endorsing TVUS, a modern assessment of TVUS's accuracy is crucial. Our research reveals a robust diagnostic performance of TVUS in detecting deep endometriosis and endometriomas and highlighting enhanced specificity for superficial endometriosis, albeit with some limitations in sensitivity. These findings empower the integration of TVUS as a primary diagnostic tool for endometriosis while emphasizing the need for further advancements in diagnostic techniques to identify cases of superficial endometriosis precisely.
